# Comment on Catanzaro et al. Risk Factors for Recurrence of Primary Sclerosing Cholangitis after Liver Transplantation: Single-Center Data. *J. Pers. Med.* 2024, *14*, 222

**DOI:** 10.3390/jpm16010054

**Published:** 2026-01-21

**Authors:** Ruslan A. Mammadov, Henk P. Roest, Maikel P. Peppelenbosch, Luc J. W. van der Laan

**Affiliations:** 1 Department of Surgery, Erasmus MC Transplant Institute, University Medical Center Rotterdam, 3015 GD Rotterdam, The Netherlands; h.roest@erasmusmc.nl (H.P.R.); l.vanderlaan@erasmusmc.nl (L.J.W.v.d.L.); 2 Department of Gastroenterology and Hepatology, Erasmus MC Transplant Institute, University Medical Center Rotterdam, 3015 GD Rotterdam, The Netherlands; m.peppelenbosch@erasmusmc.nl

Primary sclerosing cholangitis (PSC) is a chronic cholestatic liver disease characterized by progressive inflammation and fibrosis of the intra- and extrahepatic bile ducts, and is often associated with inflammatory bowel disease (IBD). Liver transplantation is the only curative treatment option, but recurrence of the disease (rPSC) occurs in approximately 15–25% of cases within 5–10 years, making this complication one of the major concerns for post-transplant outcomes. The authors identify several potential risk factors for rPSC, including donor gender, prolonged cold ischemia time, and the use of Roux-en-Y biliary reconstruction. While these findings are of clinical relevance, we would like to offer several observations and suggestions that may enhance the interpretation of the data and inform future research. Specifically, we note that associations with surgical technique and CIT may reflect confounding rather than causal factors. We also express our concern on the limited statistical power of the analysis, given the small sample size and lack of multivariable modeling. Furthermore, we highlight emerging risk factors, including host–microbiota interactions and FUT2 genetic polymorphisms, which may play a central role in rPSC pathogenesis. In our own cohort, we identified intestinal bacteremia and non-secretor FUT2 status as strong, independent predictors of rPSC. We encourage the integration of microbial and genetic markers into future risk stratification models to improve individualized post-transplant surveillance and patient outcomes.

We read with great interest the recent article by Elisa Catanzaro et al., titled “Risk Factors for Recurrence of Primary Sclerosing Cholangitis after Liver Transplantation: Single-Center Data” [1]. The authors present retrospective data on 33 patients transplanted for primary sclerosing cholangitis (PSC), with an observed recurrence rate of 27%. They identify several factors associated with recurrent PSC (rPSC), including donor gender, prolonged cold ischemia time (CIT), and the use of a Roux-en-Y biliary anastomosis. They also report that post-transplant inflammatory bowel disease (IBD) activity, including both reactivation and de novo IBD, may increase rPSC risk. We commend the authors for addressing this clinically significant and complex complication of liver transplantation. However, we would like to offer several observations and critical reflections based on our own research and existing literature, which may enhance the interpretation of the study and guide future investigations into the pathogenesis and risk stratification of rPSC.

## 1. Surgical Variables and Their Interpretability

The association between Roux-en-Y anastomosis and rPSC is potentially confounded by indication bias. Roux-en-Y is often employed in patients with complex biliary anatomy, chronic cholangitis, or in re-transplant scenarios, factors that could independently signal higher recurrence risk [2]. Without adjusting for these factors, it is difficult to determine whether the anastomotic technique itself is causally linked to recurrence or simply marks a subgroup at higher baseline risk.

## 2. Graft Cold Storage Time

Although the association between CIT and rPSC is statistically significant, any underlying mechanism remains elusive as was shown previously [3]. Prolonged CIT is a recognized as a possible risk factor for ischemic injury to the bile ducts, but its role in triggering autoimmune recurrence after liver transplantation has yet to be established [4]. A putative factor in this process is related to damage associated molecular patterns (DAMPs) as they can contribute to immune activation and inflammation by attracting neutrophils and macrophages to sites of injury, activating inflammasomes, and promoting fibrogenesis through hepatic stellate cell activation [5].

## 3. Unexpected Lack of Impact on Graft and Patient Survival

One of the most surprising findings of the study is the apparent lack of impact of rPSC on long-term graft or patient survival, contrasting prior literature where rPSC reduces graft longevity and increases re-transplant risk [6]. This contrasts with a current study suggesting that rPSC is associated with decreased graft longevity and higher risk of re-transplantation [7]. In our recent cohort study, including 81 patients who underwent liver transplantation for PSC, we found that rPSC was significantly associated with both graft failure and reduced overall survival, while showing no significant differences in CIT or sex composition [6]. Risk prediction was assessed using backward stepwise Cox proportional hazards regression, with results expressed as hazard ratios (HRs). The mean follow-up duration for the cohort was 5.8 years, ranging from 3.0 months to 20.2 years.

The discrepancy between these findings and the current study may relate to the small sample size, but could also reflect differences in rPSC case definition, follow-up duration, or post-transplant management strategies. Differences may arise from sample size, case definitions, follow-up duration, or post-transplant management. We agree with the authors that larger multicenter studies are needed to validate these findings.

## 4. Sample Size and Statistical Power

The relatively small cohort size (*n* = 33) and particularly the low number of rPSC patients (*n* = 9) raise statistical concerns. The risk of type I and type II errors is high in such small samples, and the robustness of univariate associations may be limited without multivariable adjustment. Multivariable modeling is recommended in larger, multicenter cohorts to determine independent predictors and account for confounding factors such as donor characteristics, immunosuppression, and IBD phenotype. Incorporating interaction terms between clinical and biological variables may further improve predictive accuracy.

The current study did not assess the role of microbiota and bacteremia or relevant genetic polymorphisms (e.g., FUT2, HLA, NOD2) and host-susceptibility factors. Inclusion of such parameters could have provided valuable mechanistic insights and enhanced the explanatory power of the analysis.

We suggest that integrating host–microbial and genetic markers into future studies could substantially improve recurrence prediction and enable more individualized post-transplant surveillance strategies.

Regarding microbial risk, our own cohort study identified intestinal bacteremia as a major independent risk factor for rPSC, with recurrence rates of 82% in patients with bacteremia versus 30% in those without bloodstream infections (*p* = 0.003) [6]. This finding underscores the growing recognition of the gut–liver axis and microbial translocation as key drivers of hepatic immune dysregulation. The gastrointestinal tract plays a pivotal role in PSC pathogenesis, and it is increasingly evident that microbial dysbiosis and impaired intestinal barrier integrity may facilitate disease recurrence after transplantation [8]. It would therefore be valuable for future investigations to include systematic microbiological surveillance and prospective tracking of post-transplant infectious events, particularly bloodstream infections or gut-derived sepsis, to clarify their potential role as immunological triggers for rPSC.

Regarding genetic susceptibility, the FUT2 gene, which encodes α(1,2)-fucosyltransferase and determines secretor status, plays a key role in regulating mucosal glycosylation patterns and shaping the gut microbiota. Non-secretor individuals (AA genotype of the rs601338 mutation) exhibit altered microbial composition and have been linked to increased susceptibility to autoimmune and inflammatory conditions, including PSC. In our cohort, non-secretor FUT2 carriers were significantly more likely to develop intestinal bacteremia and subsequent rPSC. Patients with both risk factors (non-secretor status plus bacteremia) demonstrated a markedly higher hazard of rPSC (HR 15.3, *p* < 0.001) [6].

These findings support a two-hit model of recurrence: (A) genetically determined microbial susceptibility and (B) an environmental or infectious trigger, such as bacterial translocation. Nonetheless, replication in larger cohorts and mechanistic validation studies are required to confirm this proposed interaction. To advance this line of research, we recommend a multicenter, prospective study design. Such a study should include systematic collection of biological samples for microbiota sequencing, blood cultures to monitor bacteremia, and genotyping for FUT2, HLA, and other relevant host susceptibility loci. Longitudinal collection of clinical and laboratory data, with structured follow-up, would allow comprehensive assessment of post-transplant outcomes, including recurrence, graft failure, and survival. Statistical analyses could employ multivariable Cox regression models with interaction terms to evaluate combined effects of microbial and genetic risk factors, facilitating precise risk stratification and identification of potential targets for intervention.

## 5. Conclusions

We commend the authors for addressing the challenging issue of PSC recurrence and for highlighting potential risk factors that contribute to ongoing discussion in this field. However, we believe that the pathogenesis of rPSC extends beyond anatomical and procedural variables and includes complex interactions between host immunity, microbial exposures, and genetic susceptibility. Expanding future investigations to incorporate host genetic factors (such as FUT2 polymorphisms), intestinal microbiota composition, and infection-related parameters may enable the development of more accurate predictive models and inform targeted preventive strategies after transplantation.
